# Difficult Conversation Case: Missed Testicular Cancer

**DOI:** 10.21980/J8.52336

**Published:** 2025-12-31

**Authors:** Joshua Ginsburg, Sarah Zamamiri, Marshall Howell, Sam Parnell, Brian Milman

**Affiliations:** * University of Texas Southwestern Medical Center, Department of Emergency Medicine, Dallas, TX

## Abstract

**Audience:**

We administered this case to senior emergency medicine (EM) residents, but it is appropriate for senior medical students and EM residents at all levels of training.

**Introduction:**

The practice of emergency medicine regularly requires navigating challenging conversations and delivering difficult news. The way physicians interact with patients in these cases can significantly influence outcomes, including the patient’s understanding of their diagnosis and treatment plan, satisfaction with care, and willingness to follow medical advice. It is therefore imperative that emergency medicine residency training emphasizes communication skills, which will also be tested on the new American Board of Emergency Medicine (ABEM) Certifying Exam.^1^

**Educational Objectives:**

This difficult conversation case is intended to assess the examinee’s ability to disclose sensitive, unexpected information to a patient regarding a missed diagnosis of testicular cancer. By the end of this session, learners should be able to, 1) demonstrate effective communication, including establishing rapport, acknowledging a prior misdiagnosis, and disclosing a revised diagnosis of cancer, 2) elicit and react to the patient’s emotional and informational needs in an empathetic and professional manner, and 3) convey a patient-centered plan of care, including appropriate next steps and coordination with specialist services.

**Educational Methods:**

We created a 10-minute case in the style of an Objective Structured Clinical Exam (OSCE) requiring resident examinees to break bad news. The case was revised after pilot testing on two additional faculty members. Materials included a task sheet for examinees based on example Certifying Exam materials provided by ABEM, a script for examiners with specific attention to eliciting elements of the SPIKES and NURSE frameworks, and a scoring sheet. The faculty who created the case then served as examiners during a Mock Certifying Exam Day for PGY-3 Emergency Medicine residents; alternatively, this case can be run with a standardized patient. For the remainder of this case, we will refer to the person playing the role of patient as “examiner.”

**Research Methods:**

Residents were evaluated using a 15-point rubric, with a score of 11/15 (73%) required to pass. The rubric, which was developed based on the objectives for Difficult Conversations Cases published on the ABEM website, included the following categories: establish rapport, determine baseline knowledge, disclose information, respond and react appropriately, and provide closure. Consistent with our program’s usual OSCE workflow, each resident was evaluated by a single faculty examiner. After completing the case, each resident completed an anonymous two-item evaluation: The first item, “This case increased my understanding of the certifying exam format,” was scored on a 5-point Likert scale from “strongly disagree” to “strongly agree.” The second item, “How would you rate the overall quality of this case?” was scored on a 5-point Likert scale from “poor” to “excellent.” The survey and protocol were reviewed by our institutional IRB on 12/3/2024, and this project was determined not to meet the definition of human subjects research.

**Results:**

Seventeen PGY-3 emergency medicine residents completed the case, with a mean score of 13.35/15. Seventeen residents (100%) completed the post-case evaluation. When asked if this case increased understanding of the certifying exam format, 17 (100%) agreed or strongly agreed. When asked about the overall quality of the case, 17 (100%) said either very good or excellent. The case received a score of 4.82/5 for overall quality.

**Discussion:**

This case was effective, as evidenced by the results that all residents agreed or strongly agreed that the case increased their understanding of the certifying exam content, and all residents considered the case quality to be very good or excellent. Residents overall performed well on the case but may benefit from additional instruction on disclosure of sensitive, unwanted, or unexpected information. Specifically, residents should be taught to use the SPIKES (Setting, Perception, Invitation, Knowledge, Emotions/Empathy, Strategy/Summary) or GRIEV_ING (Gather, Resources, Identify, Educate, Verify, Give Space, Inquire, Nuts and Bolts, Give) frameworks for breaking bad news and NURSE (Naming, Understanding, Respecting, Supporting, Exploring) statements for responding to emotions.^2^–^4^ This case and its grading rubric could easily be adapted to other difficult conversation scenarios to prepare emergency medicine residents or graduates for their certifying exam. However, because each resident was evaluated by a single examiner within one residency program, inter-rater reliability could not be assessed, and generalizability may be limited.

**Topics:**

Difficult conversations, breaking bad news, communication, certifying exam preparation.

**Table:** 

List of Resources:	
Abstract	1
User Guide	3
For Examiner Only	5
Certifying Exam Assessment	9
Stimulus	10
Debriefing and Evaluation Pearls	12

**Learner Audience:**

This case is appropriate for medical students, interns, and junior and senior residents.

**Time Required for Implementation:**

Case: 10 minutes

Debriefing: 5 minutes

**Recommended number of learners per instructor:**

one learner per instructor

**Topics:**

Difficult conversations, breaking bad news, communication, certifying exam preparation.

**Objectives:**

By the end of this session, learners should be able to:

1. Demonstrate effective communication, including establishing rapport, acknowledging a prior misdiagnosis, and disclosing a revised diagnosis of cancer
2. Elicit and react to the patient’s emotional and informational needs in an empathetic and professional manner
3. Convey a patient-centered plan of care, including appropriate next steps and coordination with specialist services.

## Linked objectives, methods and results

This OSCE format is well-suited to achieve the objectives because it is a structured, time-limited environment that allows examinees to demonstrate key communication competencies under the same conditions that will be present during their certifying exam. The patient in this case is initially upset about being called back to the emergency department without explanation. Examinees must quickly establish rapport with the patient to de-escalate the situation and allow for effective disclosure of error and the new diagnosis of cancer. The script then includes realistic reactions and questions from the patient that require the examinee to empathetically elicit and react to the patient’s emotional and informational needs. Finally, the case leads naturally to a conclusion that necessitates clear communication of a plan of care and next steps. The SPIKES mnemonic, which is cited in the reference material from ABEM, was used as a framework to inform the development of this content and objectives. Meeting the objectives ensures that the examinee accomplishes the five requirements outlined by ABEM: establish rapport, determine baseline knowledge, disclose information, respond and react appropriately, and provide closure.

## Recommended pre-reading for instructor

- American Board of Emergency Medicine. Certifying Exam Content. Accessed November 18, 2025. https://www.abem.org/get-certified/certifying-exam/certifying-exam-content/
- Baile WF, Buckman R, Lenzi R, Glober G, Beale EA, Kudelka AP. SPIKES-A six-step protocol for delivering bad news: application to the patient with cancer. *Oncologist.* 2000;5(4):302–11. PMID: 10964998. doi: 10.1634/theoncologist.5-4-302
- Hobgood C, Harward D, Newton K, Davis W. The educational intervention “GRIEV_ING” improves the death notification skills of residents. *Acad Emerg Med.* 2005;12(4):296–301. PMID: 15805319. doi: 10.1197/j.aem.2004.12.008
- Back A, Arnold R, Tulsky J. *Mastering Communication with Seriously Ill Patients: Balancing Honesty with Empathy and Hope.* Cambridge University Press; 2009.

## Results and tips for successful implementation

We implemented this case during a Mock Certifying Exam Day for PGY-3 Emergency Medicine residents, which allowed for an experience that closely resembled the environment of the actual exam. However, this case could easily be utilized in isolation rather than as part of a structured day of examinations. The case was conducted in an examination room with the examiner sitting on the examination chair to simulate an emergency department environment as accurately as possible and to make it clear that the examiner is playing the role of the patient. However, this case could be delivered virtually or in a room without an examination chair as long as roles are made clear.

We created a rubric applicable to many difficult conversation cases. The rubric was reviewed and modified by two other faculty members involved in this project, and then pilot-tested on two additional faculty members, leading to minor changes in the wording of anchors prior to use. We tested 17 PGY-3 emergency medicine residents and immediately evaluated their performance after case completion. The mean resident performance score was 13.35/15. The lowest scoring category was "disclose information,” and the highest scoring category was "provide closure." Seventeen residents (100%) completed the post-case evaluation. When asked if this case increased understanding of the certifying exam format, 17 (100%) agreed or strongly agreed. When asked about the overall quality of the case, 17 (100%) said either very good or excellent. The case received a score of 4.82/5 for overall quality.

Because informal feedback from the post-case debrief was also positive, we did not make any modifications to this case after implementation.

## Figures and Tables

**Table:** Difficult Conversations Candidate Task Sheet

**CASE PARAMETERS**	
This is a 10-minute case. You will interact with a patient. You will discuss the diagnosis of testicular cancer, which was initially misdiagnosed as “nonspecific orchitis” when the patient presented to the ED last night with left testicular pain and swelling. Answer the patient’s questions and explain next steps.	
**PATIENT INFORMATION**	
**Patient name**	John Wells
**Age**	29 y/o
**Gender identity**	Man
**Presenting complaint**	Left testicular pain, Results Callback
**General appearance/History of present illness**	Testicular pain x one month. He was here yesterday evening and had a discharge diagnosis of orchitis. He was called back this morning and told to return to the ED.
**Past medical history**	None
**Medication**	None
**Allergies**	None
**Vital signs on ED arrival**	Normal
**RESULTS/FINDINGS**	
The patient initially presented to the ED yesterday evening with left testicle pain and swelling for one month. The laboratory results and urinalysis were unremarkable, and the preliminary scrotal ultrasound was read by the radiology resident as "nonspecific orchitis.” The patient was sent home with oral antibiotics and supportive care. This morning, the attending radiologist overread the ultrasound as “Irregular testicular mass highly suggestive of malignancy.” Your colleague left a voicemail telling the patient to return to the ED but did not provide more information.	
**TASK STATEMENT**	
Your task is to inform Mr. Wells about the change in his ultrasound reading and discuss the likely diagnosis of testicular cancer.	

## FOR EXAMINER ONLY

### Difficult Conversations Case: Missed Testicular Cancer Summary

**Diagnosis:** Testicular cancer

**Case Summary:** This 10-minute case will test the examinee’s ability to have a difficult conversation. As the examiner, you will be playing the role of the patient. Alternatively, this case can be run with a standardized patient. The examinee will be breaking bad news regarding likely testicular cancer that was initially misdiagnosed as orchitis based on a preliminary ultrasound reading from an ED visit the previous night. High-performing examinees are expected to establish rapport, determine baseline knowledge, disclose information, respond and react appropriately, and provide closure.

**Standardized Actor Profile:** You are playing the role of the patient, John Wells, a 29-year-old, previously healthy man. You have a high school education and work in construction. You have health insurance, but your medical literacy is limited. You have a wife but no kids, although you would like to have biological children soon.

**Materials/personnel needed:**

One examiner or standardized patient is needed. No props are necessary.

**Room Setup:**

One examination bed for the standardized patient to sit on (ideal but not necessary).

**Play of Case** ***:***

In an ideal play of the case, the encounter begins with the examiner expressing appropriate frustration about the long wait time, the unexpected callback, and the lack of information provided, creating an opportunity for the examinee to acknowledge the patient’s experience, apologize, and establish rapport. As the conversation progresses and the examinee seeks to understand the patient’s baseline knowledge, the examiner should focus on the prior, more benign diagnosis of orchitis that was given the previous evening. When the examinee discloses the revised ultrasound findings and explains the concern for testicular cancer, the examiner should initially respond with disbelief and confusion, and then transition to asking clarifying questions about prognosis, fertility, and next steps. This allows the examinee to demonstrate empathic communication, provide clear explanations, and respond to emotional cues using frameworks such as SPIKES and NURSE. As the encounter approaches its conclusion, if the examinee has not yet addressed follow-up or the care plan, the examiner should ask about next steps, such as involvement of urology or anticipated imaging, which should prompt the examinee to summarize the plan, ensure understanding, and provide a clear sense of closure. Throughout the case, the conversation should unfold naturally, with the examiner using the provided prompts to support an interaction that reflects the key behaviors expected during a difficult conversation OSCE.

### Difficult Conversations Case: Missed Testicular Cancer Standardized Patient Script

#### Key Point 1: Establishing Rapport

1. **REQUIRED STATEMENT: “Hello Doctor, you will have 10 minutes to complete this case. You may review the task sheet and begin.”**
2. **Immediately after the examinee introduces himself/herself, the examiner should express frustration about the long wait time and lack of information.**
  ○ “Are you my doctor?”
  ○ “What on earth is going on? I was sent home after six hours last night then told to come back this morning without any more information! And I just sat in the waiting room for three more hours with no answers! What kind of hospital is this?”
  ○ “Tell me why I had to wait so long!”
3. **Examinee will likely apologize for the wait and/or callback. Continue to express frustration but move the conversation along.**
  ○ “It’s just ridiculous. I’m in pain, and I’m already late for work. Let’s hurry up.”

#### Key Point 2: Assessing Baseline Knowledge

4. **If initially asked “What do you think is going on?” or “What do you know about what’s going on?” the examiner should focus on the previous, incorrect diagnosis**
  ○ “Well, they put ‘orchitis’ on my discharge paperwork, although I still don’t really know what that is. Inflammation, I guess?”
  ○ “They said the antibiotics should work, so I’m fine with that.”

#### Key Point 4: Answering Questions

6. **After further explanation from the examinee, begin to accept the diagnosis and ask questions**
  ○ “OK. Well, what does that mean for me? I know there are good and bad cancers… Is it bad cancer?”
  ○ "Has it already spread? Am I going to die?"
  ○ “I want to have kids this year. Can I still have kids?”

#### Key Point 5: Ensuring Closure and Next Steps

7. **As the 10-minute mark approaches, if not yet addressed, begin to move the conversation to a close by asking questions that transition to a conclusion.**
  ○ “Can I call my family so you can explain this?”
  ○ “Do I need to be admitted to the hospital?”
  ○ “Is my insurance going to cover this? Who can I talk to about that?”
8. **To conclude, show appreciation:**
  ○ “OK. Thank you for explaining everything. What do I do now?”
  ○ “I appreciate your help doctor. I’ll do whatever is necessary.”

## CERTIFYING EXAM ASSESSMENT

*Difficult Conversation Case: Missed Testicular Cancer*

Learner: _________________________________________

**Establish rapport with patient**

□ Introduce self as “doctor”
□ Identify patient
□ Maintain eye contact

**Ascertain the baseline state of knowledge regarding the situation**

□ Use open-ended questions to understand patient’s current understanding

**Disclose the sensitive information**

□ Be concise
□ Use plain language
□ Acknowledging the prior misdiagnosis and disclose the revised diagnosis of cancer

**Respond and react accordingly**

□ Support the patient
□ Convey a patient-centered plan of care, focusing on involvement of specialist services. Either immediate consultation with or prompt referral to urology is appropriate to achieve this critical action because institutional guidelines and services vary.

**Provide closure to conversation**

□ Answer questions
□ Clarify next steps

**Summative and formative comments:**

### Stimulus Inventory

**Candidate Task Sheet**

## DEBRIEFING AND EVALUATION PEARLS

### Missed Testicular Cancer

*Structured debrief guide (should be accomplished in about five minutes)*

1. **Opening:**
  - Ask the learner, “How did that feel?”
  - Normalize any reported difficulty with a statement such as, “These conversations are inherently challenging, even for seasoned clinicians.”
2. **Provide focused feedback based on the rubric:**
  - Rapport: Did the learner state their name and role, speak clearly, sit down, make eye contact, and use open body language?
  - Baseline knowledge: Did the learner elicit the patient’s understanding of his prior diagnosis?
  - Disclosure: Did the learner use a warning shot, acknowledge the misdiagnosis, and avoid jargon?
  - Responding to Emotions: Use of empathy, supportive presence, use of NURSE statements.^4^ Examples of NURSE statements specific to this case are below:
    ○ Naming: “It sounds like you’re really worried.”
    ○ Understanding: “I can understand why this is confusing, especially after being told something different yesterday.”
    ○ Respecting: “You’re asking important questions. That kind of clarity helps us make sure you get the right care.”
    ○ Supporting: “I want you to know that we will make sure you are supported through every step of this.”
    ○ Exploring: “What is your biggest concern now?”
  - Closure: Explanation of the next steps and ensuring all questions were asked and answered.
3. **Teaching points:**
  *Communication teaching points*
  - Provide concrete example phrasing using SPIKES and NURSE that could strengthen the learner’s approach. SPIKES examples specific to this case are below:^2^
    ○ Setting: “I can see this has been a stressful morning. Let’s sit down and talk openly about what we found and what it means.”
    ○ Perception: “Can you tell me what you understand so far about what brought you back today?”
    ○ Invitation: “Would it be OK if I shared the updated results of your ultrasound now?”
    ○ Knowledge: “I’m afraid I have bad news. The updated ultrasound shows a mass in your testicle that is very concerning for cancer.”
    ○ Emotions/Empathy: “I can see this is shocking. Anyone in your situation would feel that way. I’m here with you, and we’ll talk about the next steps.”
    ○ Strategy/Summary: “The next steps are to involve our urology team and discuss additional tests. I will be back to talk to you as soon as I speak to them. Please let a nurse know if you have any questions before then, and I’ll come answer them.”
  - Reinforce transferability: “This framework applies similarly to other cases of unexpected or difficult diagnoses.”

*Testicular cancer teaching points:*

Testicular cancer is the most common solid malignancy in young men. Typically, it presents as a painless testicular mass. Key steps after suspicion include scrotal ultrasound and prompt referral to urology for further evaluation, including serum tumor markers (AFB, β-hCG, LDH), and planning for radical orchiectomy, which is both diagnostic and therapeutic. The specific timing of urology evaluation may vary depending on clinical stability and institutional resources, but clinicians should ensure that follow-up is clearly arranged. Because early-stage disease has a 99% 5-year survival rate, rapid recognition and timely referral are critical.^5^
